# Emerging Hypervirulent Marek’s Disease Virus Variants Significantly Overcome Protection Conferred by Commercial Vaccines

**DOI:** 10.3390/v15071434

**Published:** 2023-06-25

**Authors:** Jin-Ling Liu, Man Teng, Lu-Ping Zheng, Feng-Xia Zhu, Shu-Xue Ma, Lin-Yan Li, Zhi-Hui Zhang, Shu-Jun Chai, Yongxiu Yao, Jun Luo

**Affiliations:** 1 Key Laboratory of Animal Immunology, Ministry of Agriculture and Rural Affairs of China & Henan Provincial Key Laboratory of Animal Immunology, Henan Academy of Agricultural Sciences, Zhengzhou 450002, China; 2 UK-China Centre of Excellence for Research on Avian Diseases, Henan Academy of Agricultural Sciences, Zhengzhou 450002, China; 3 Zhumadian Center for Animal Disease Control and Prevention, Zhumadian 463000, China; 4 Suiping Center for Animal Disease Control and Prevention, Zhumadian 463100, China; 5 College of Animal Science and Technology, Henan University of Science and Technology, Luoyang 471003, China; 6 The Pirbright Institute & UK-China Centre of Excellence for Research on Avian Diseases, Pirbright, Ash Road, Guildford GU24 0NF, Surrey, UK

**Keywords:** Marek’s disease, MDV, pathogenicity, oncogenicity, vaccine, protection indices

## Abstract

As one of the most important avian immunosuppressive and neoplastic diseases, Marek’s disease (MD), caused by oncogenic Marek’s disease virus (MDV), has caused huge economic losses worldwide over the past five decades. In recent years, MD outbreaks have occurred frequently in MD-vaccinated chicken flocks, but the key pathogenic determinants and influencing factors remain unclear. Herein, we analyzed the pathogenicity of seven newly isolated MDV strains from tumor-bearing chickens in China and found that all of them were pathogenic to chicken hosts, among which four MDV isolates, SDCW01, HNXZ05, HNSQ05 and HNSQ01, were considered to be hypervirulent MDV (HV-MDV) strains. At 73 days of the virus infection experiment, the cumulative incidences of MD were 100%, 93.3%, 90% and 100%, with mortalities of 83.3%, 73.3%, 60% and 86.7%, respectively, for the four viruses. The gross occurrences of tumors were 50%, 33.3%, 30% and 63.3%, respectively, accompanied by significant hepatosplenomegaly and serious atrophy of the immune organs. Furthermore, the immune protection effects of four commercial MD vaccines against SDCW01, CVI988, HVT, CVI988+HVT, and 814 were explored. Unexpectedly, during the 67 days of post-virus challenge, the protection indices (PIs) of these four MD vaccines were only 46.2%, 38.5%, 50%, and 28%, respectively, and the birds that received the monovalent CVI988 or HVT still developed tumors with cumulative incidences of 7.7% and 11.5%, respectively. To our knowledge, this is the first demonstration of the simultaneous comparison of the immune protection efficacy of multiple commercial MD vaccines with different vaccine strains. Our study revealed that the HV-MDV variants circulating in China could significantly break through the immune protection of the classical MD vaccines currently widely used. For future work, there is an urgent need to develop novel, more effective MD vaccines for tackling the new challenge of emerging HV-MDV strains or variants for the sustainable control of MD.

## 1. Introduction

Marek’s disease (MD) is a significant avian disease caused by Marek’s disease virus (MDV), and it can lead to severe immunosuppression, T-cell lymphomas, and nerve system symptoms in poultry [1]. MD seriously harms the global poultry industry, causing huge direct economic losses of more than USD 1 billion annually [2]. MDV is classified in the genus *Mardivirus*, belonging to the subfamily *Alphaherpesvirinae* of *Herpesviridae* [3]. MDV possesses a large DNA genome of about 180 kb in length and encodes more than one hundred viral protein-coding genes and non-coding RNA genes, which result in complex pathogenesis and tumorigenesis during the course of the disease [4,5,6]. Historically, according to its antigenicity, MDV has been divided into three serotypes, including serotype 1 (MDV-1), serotype 2 (MDV-2), and serotype 3 (MDV-3)/herpesvirus of turkeys (HVT), which have been reclassified as *Gallid alphaherpesvirus* 2 (GaAHV-2), *Gallid alphaherpesvirus* 3 (GaAHV-3), and *Meleagrid alphaherpesvirus* 1 (MeAHV-1) in the latest refreshed virus taxonomy [3]. Among the three serotypes of MDV, only the virulent MDV-1 strains are pathogenic and/or oncogenic to chicken hosts. In the past 50 years, the long-term and high-intensity vaccine immune pressure has driven the persistent evolution and increased virulence of MDV [7]. According to the different pathogenicity and virulence levels evaluated with monovalent HVT or bivalent HVT+SB-1 vaccinations, MDV-1 isolates have been further divided into four pathotypes: mild MDV (mMDV), virulent MDV (vMDV), very virulent MDV (vvMDV), and very virulent plus MDV (vv+MDV) [7,8]. However, some of the virulent variants, especially hypervirulent MDV (HV-MDV) strains recently isolated from clinical cases of MD vaccinated chicken flocks in China, could be designated as HV-MDV variants [9,10], based on significantly different genetic evolution routes displayed by independent genetic evolution branches in phylogenetic trees generated with the major oncogene *meq* or the whole viral genomic sequences [11,12,13,14].

MD is a virally induced immunosuppressive and neoplastic disease that can be effectively prevented and controlled with antiviral vaccines, which have played a crucial role in the efficient control of the disease in poultry. At present, the most widely used classical vaccine strains for commercial MD vaccine productions are HVT FC-126 [15] for serotype 3, SB-1 [16] for serotype 2, and CVI988/Rispens (CVI988) [17,18], or 814 [19] for serotype 1. The utilization of these vaccine strains alone or in combination has contributed significantly to reducing outbreaks and the prevalence of MD around the world [20,21,22]. However, although the use of MD vaccine can effectively prevent the disease, including the occurrence of tumors, it cannot completely block the infection and transmission of MDV. High immune pressure or imperfect vaccination can drive the evolution and enhancement of the virulence of MDV [7,23]. This makes the commonly used classical MD vaccines unable to provide effective protection against the emerging vvMDV and vv+MDV strains and/or HV-MDV variants. In recent years, several countries around the world have reported the occurrences of MD in many chicken flocks that have been vaccinated with MD vaccines [24]. The emerging strains of vvMDV, vv+MDV, and other HV-MDV variants are thought to be the major reasons for MD vaccine failures.

As one of the largest poultry breeding bases in the world, in China, there are 14–16 billion birds need to be vaccinated annually against MD. In the past two decades, epidemics and outbreaks of MD have frequently happened in MD-vaccinated poultry farms in most regions in China [9,10,11,12,13,25,26,27]. In recent years, especially in the period of 2019–2023, a large number of poultry farms and chicken flocks have suffered from serious tumor-bearing diseases, in particular from the south to the northeast of China and especially in the central–eastern region. Thus, in a recent study [28], we performed an epidemiological investigation and differential diagnosis on a total of 30 chicken flocks from 42 tumor-bearing poultry farms in central China and revealed that this wave of avian neoplastic diseases was mainly caused by the prevalence and infection of MDV. To further explore the pathogenicity of the latest epidemic MDV strains and comprehensively evaluate the immune protection efficacy of MD vaccines, we carried out a pathogenicity analysis of seven new MDV isolates and simultaneously evaluated the immune protection effects of the four MD vaccines most widely used in China. Our data suggested that the HV-MDV variants are widely prevalent in Chinese chicken flocks, and some of them have significantly broken through the immune protection of classical MD vaccines, bringing a new challenge for MD control in the future.

## 2. Materials and Methods

### 2.1. Viruses and Cells

Seven MDV isolates, namely, SDCW01, HNXZ05, HNSQ05, HNSQ01, HNKF01, HNXZ01, and HNFQ03, were isolated from the clinical MD cases recently reported in central China, as previously described [12,28]. The detailed background of these MDV isolates is shown in Table 1. Four commercial MD vaccines, including live cell vaccines CVI988, CVI988+HVT, 814, and freeze-dried vaccine HVT, were provided by corresponding agents from the market in Henan province. The primary chicken embryo fibroblast (CEF) monolayers were prepared from 9-day-old SPF chicken embryos (provided by Beijing Boehringer Ingelheim Vital Biotechnology Co., Ltd., Beijing, China). MDV proliferation and viral titer titration were performed with IFA staining and viral plaque counting, as previously described [29,30,31].

### 2.2. Chickens

One-day-old specific pathogen-free (SPF) White Leghorns, a Babcock pure line with the B-haplotype originally introduced from AVS Bio (Avian Vaccine Services, which used to belong to Charles River Laboratories before 2022, Norwich, CT, USA) and bred by Spirax Ferrer Poultry Science and Technology Co. Ltd. (Jinan, China), were kept in positive pressure chicken isolators. The birds were fed normally according to the nutritional requirements of different ages.

### 2.3. Animal Experiments for Pathogenicity Study

To evaluate the pathogenicity and oncogenicity rate of MDV isolates, a total of 381 one-day-old SPF chickens were randomly divided into 8 groups, in which 48 birds were allocated to groups 1–7, and another 45 birds were placed in group 8, which served as the CEF mock infection group. Birds in groups 1–7 were separately injected with the CEF-grown MDV stocks of SDCW01, HNXZ05, HNSQ05, HNSQ01, HNKF01, HNXZ01, or HNFQ03 viruses, via intraperitoneal inoculation in a blinded manner and with 2000 plaque formation units (PFUs) for each bird. The birds in group 8 were individually inoculated with an equal volume of mock CEF suspension and served as a negative control. Post-infection, the diseased and dead birds were each examined and dissected for the observation of gross pathological changes to record the occurrence of tumors. At 73 days post-infection (dpi), survivors from each group were humanely euthanized for the observation and recording of tumor occurrence.

### 2.4. Vaccination and Virus Challenge Experiments

In order to evaluate the immune protection efficacy of different MD vaccines against MDV isolate SDCW01, a total of 216 one-day-old SPF chickens were randomly divided into 6 groups (*n* = 36/group). Equal volumes of CEFs were subcutaneously injected into the back of the neck of birds in mock infection groups 1 and 2, whereas birds in groups 3–6 were vaccinated with four commercial MD vaccine products: CVI988, HVT, CVI988+HVT, and 814, respectively, in a blinded manner. One dose per bird was used for three live-cell vaccines, CVI988, CVI988+HVT, and 814, according to the product manuals, whereas five doses of the freeze-dried HVT vaccine were used for each bird. Subsequently, at 7 days of age, birds in group 1 were intraperitoneally inoculated with an equal volume of CEFs to serve as a negative control, whereas birds in groups 2–6 were challenged with the MDV isolate SDCW01 (1000 PFUs/200 μL per bird). Post-challenge, the birds were observed daily, and postmortem examinations of the dead chickens were carried out for the observation of gross pathological changes and the occurrence of tumors. At 67 days post-challenge (dpc), the surviving chickens in each group were humanely euthanized, and the tumor occurrence was recorded.

### 2.5. Determination of Body and Immune Organ Weights

Post virus infection or challenge, five birds were randomly selected from each group for weighing at one-week intervals. At 14 and 21 dpi/dpc, five birds from each group were humanely euthanized, and the immune organs, including the thymus, bursa, and spleen, were collected and weighed. The average and standard deviation of body weight of birds from each group were calculated and compared to the mock controls, and the immune organ indices of virus-infected or challenged birds were also calculated using the following formula: (immune organ mass/body mass) × 100.

### 2.6. Statistical Analysis

Deaths that happened within one-week post-virus infection in the first batch of animal experiments or post-MD-vaccination in the second batch of animal experiments were excluded from the total numbers. GraphPad Prism Version 6.0 (GraphPad Software, Inc., San Diego, CA, USA) was used to statistically analyze the means and standard deviations (M ± SD) of body weight and immune organ indices of virus-infected/challenged chickens and to draw the survival curves. The statistical significances of body weight or immune organ indices between different groups were compared using two-way ANOVA (Tukey’s multiple comparisons test, with alpha = 0.05). MD incidence rate = {(number of deaths + number of survivals with clinical symptoms and/or gross tumors)/total number of infected birds} × 100%. The immune protection index (PI) was calculated as PI = {(% MD of unvaccinated chickens − % MD of vaccinated chickens)/% MD of unvaccinated chickens} × 100%. The statistical significances of morbidity, mortality, gross tumor incidence, and PIs between different groups were compared using Z test. The differences in survival patterns between each virus-infected/challenged group were statistically analyzed using a Log-rank (Mantel–Cox) test (GraphPad Prism Version 6.0).

## 3. Results

### 3.1. Growth Rates and Immunosuppression of Birds Infected with Seven MDV Isolates

**Figure 1:**
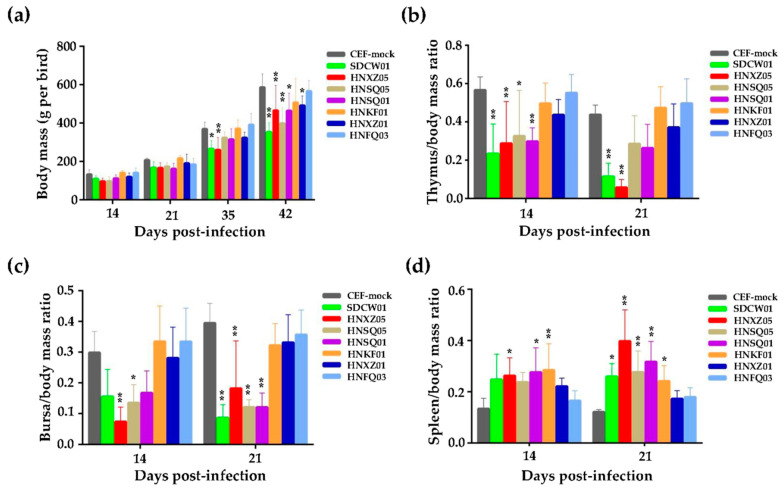
Growth rates and the ratios of thymus, bursa or spleen over body mass of chickens infected with seven distinct MDV isolates. (**a**) Growth rates of virus-infected birds. (**b**–**d**) Ratios of thymus, bursa, or spleen over body mass of virus-infected birds. For each group, the thymus masses, bursa masses, and spleen masses of five randomly selected birds were measured at 14- and 21-days post-infection (dpi). From then on, the body masses of five randomly selected birds from each group were measured at 14, 21, 35, and 42 dpi. The data are shown as M ± SD (*n* = 5), and the statistical significance compared to the CEF mock infection controls is shown by single or double stars (*p* < 0.05 or *p* < 0.01, respectively). The error bars indicate the standard deviations of body mass and immune organ indices.

Seven newly isolated MDV viruses from central China, namely, SDCW01, HNXZ05, HNSQ05, HNSQ01, HNKF01, HNXZ01, and HNFQ03, as listed in Table 1, were used to perform the animal experiments using 1-day-old SPF chickens. Samples of five randomly selected birds from each group were taken at 14 and 21 dpi for the statistical analysis of body mass and immune organ index. Compared to the CEF mock infection control, as shown in Figure 1a, no significant differences in the body mass of all virus-infected groups were observed at 14 or 21 dpi. With the prolongation of virus infection, e.g., at 35 dpi, the body masses of chickens in most virus-infected groups were lower than those in the CEF mock infection group. In particular, at 42 dpi, the body masses of birds in all virus-infected groups were significantly lower than those of the birds in the CEF mock infection group (*p* < 0.05 or *p* < 0.01), except for those of the HNKF01- or HNFQ03-infected birds.

At 14 and 21 dpi, the thymus/body mass ratios of most of the seven virus-infected groups were lower than those of the CEF mock infection group, and four groups infected with SDCW01, HNXZ05, HNSQ05, and HNSQ01 displayed significant differences from the CEF mock infection group (Figure 1b). The data of the bursal index were very similar to the thymus index, especially for SDCW01-, HNXZ05-, HNSQ05-, and HNSQ01-infected groups, and were also significantly lower than the CEF mock group measured at 14 or 21 dpi (Figure 1c). Inversely, for the spleen indices, the spleen/body mass ratios of birds in all virus-infected groups were higher than those in the CEF mock infection group, and the ratios in birds in five groups infected with SDCW01, HNXZ05, HNSQ05, HNSQ01, or HNKF01 were all significantly higher than those birds in the CEF mock infection group (Figure 1d).

### 3.2. Variable Pathogenicities and Oncogenicities of Seven MDV Strains

Except for the birds sacrificed for sample collection at 14 and 21 dpi (*n* = 10) and non-specific deaths (*n* = 8, 8, 8, 8, 5, 9, 8, and 9 in groups 1–8, respectively) that were possibly caused by the adverse effects of virus infection in the first week, all the dead and surviving birds at 73 days were checked to evaluate the pathogenicity and oncogenicity of the virus, including the rates of cumulative MD morbidity, mortality, and tumor incidence. As shown in Table 2, both SDCW01 and HNSQ01 were displayed as the most virulent strains, with high MD incidence rates (100% and 100%), mortalities (83.3% and 86.7%), and tumor occurrence rates (50% and 63.3%), followed by the strains of HNXZ05 and HNSQ05, with MD incidence rates of 93.3% and 90%, mortalities of 73.3% and 60%, and tumor occurrence rates of 33.3% and 30%, respectively. The strains of HNKF01 and HNFQ03 were less virulent, with relatively lower MD incidence rates (45.5% and 34.5%), mortality (21.2% and 34.5%), and tumor occurrence rates (24.2% and 0%), respectively. The strain of HNXZ01 showed the weakest virulence, of which the MD incidence, mortality, and tumor occurrence were 10%, 10%, and 0%, respectively. For five oncogenic MDV strains, the gross tumors induced by SDCW01, HNXZ05, HNSQ01, and HNKF01 were mainly present in the liver and/or spleen of surviving birds, whereas those induced by HNSQ05 were scattered across multiple organs, including the liver, spleen, kidney, and heart.

**Figure 2:**
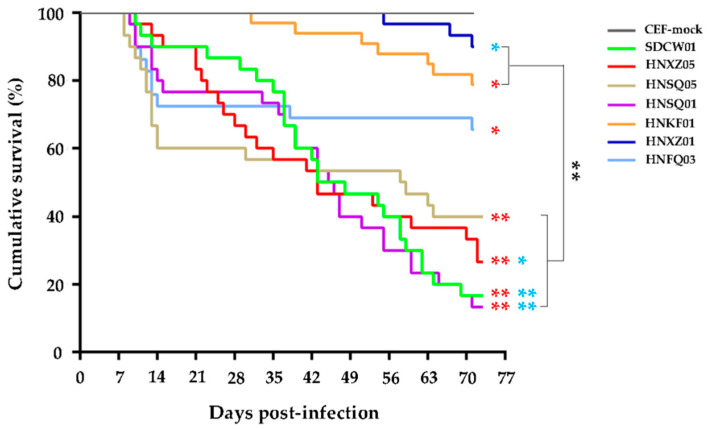
Survival curves of chickens infected with seven distinct MDV strains over the 73-day experimental time period. For each group, the birds were individually infected with CEFs containing 2000 PFU viruses via intra-abdominal inoculation, whereas, for the negative controls, the birds were inoculated with an equal volume of mock CEFs. Early deaths, possibly due to adverse effects of virus inoculation, and birds sacrificed for body weighting and immune organ collections were excluded for data calculation. The differences in survival patterns between each virus-infected group were statistically analyzed using a Log-rank (Mantel–Cox) test (GraphPad Prism Version 6.0). The significance of virus-challenge groups compared to CEF mock infection controls are shown by single or double red stars (* *p* < 0.05 or ** *p* < 0.01). Significant differences between HNFQ03 and the other virus-challenge groups are shown by blue stars, whereas those between four highly virulent MDV isolates and the two lower virulent viruses are shown by black brackets and stars.

The survival curves in each group were drawn according to the daily recorded deaths and mortalities of virus-infected birds, and the data were basically similar to those described above. As shown in Figure 2, although the onset of disease and the tendency towards death induced by seven MDV strains post-infection were quite different, the birds’ deaths caused by the four most virulent MDV strains, including SDCW01, HNSQ01, HNXZ05 and HNSQ05, were very quick, and this occurred continuously throughout the whole experimental time period. As for the other three MDV strains, the deaths of HNFQ03-infected birds were mainly at an early stage of the disease, whereas deaths caused by HNKF01 and HNXZ01 infections occurred more frequently at the mid or late stages of the disease (Figure 2). Except for HNXZ01, as demonstrated in Figure 2, the death patterns in birds caused by SDCW01, HNSQ01, HNXZ05, HNSQ05, HNFQ03, or HNKF01 were significantly different from the CEF mock infection controls (*p* < 0.01 or *p* < 0.05). No significant difference was observed among the four highly virulent MDV strains (SDCW01, HNSQ01, HNXZ05, and HNSQ05) or among the other two viruses with lower virulence (HNFQ03 or HNKF01). However, compared to the three viruses with lower virulence, four highly virulent MDV strains caused significant severe death patterns (*p* < 0.01). Accumulatively, the survival rates of birds infected with SDCW01, HNSQ01, HNXZ05, or HNSQ05 were 16.7%, 13.3%, 26.7%, and 40%, respectively, lower than those of the other three groups infected with HNFQ03, HNKF01, or HNXZ01 (65.5%, 78.8%, and 90%, respectively) over the 73-day experimental time period. The median survival rates of SDCW01-, HNSQ01-, HNXZ05-, or HNSQ05-infected birds were 45.5 day, 45.5 day, 43 day, and 58.5 day, respectively.

### 3.3. Growth Rates and Lymphoid Organ Weights of Distinct MD-Vaccinated Birds Challenged by the SDCW01 Strain

**Figure 3:**
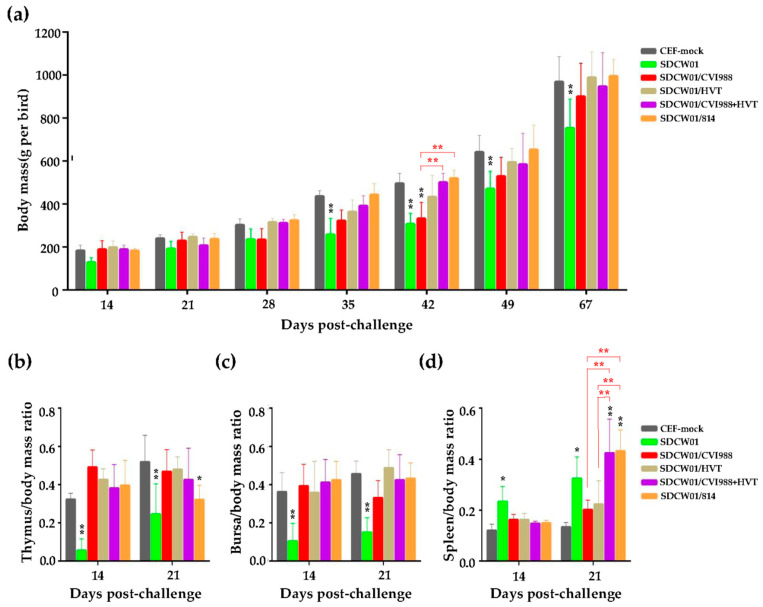
Growth rates and the ratios of thymus, bursa or spleen over body mass of distinct MD-vaccinated birds challenged by SDCW01 strain. (**a**) Growth rates of distinct MD-vaccinated birds challenged by SDCW01 strain. (**b**–**d**) Ratios of thymus, bursa, or spleen over body mass of distinct MD-vaccinated birds challenged by SDCW01 strain. For each group, the thymus mass, bursa mass, and spleen mass of five randomly selected birds were measured at 14 and 21 dpc, and the body masses of five randomly selected birds were measured at 14, 21, 28, 35, 42, 49, and 67 dpc, respectively. The data are shown as M ± SD (*n* = 5), and the error bars indicate the standard deviation of body mass and immune organ indices. The black stars indicate significant differences (* *p* < 0.05 or ** *p* < 0.01) compared to the CEF mock controls, whereas the significant differences among distinct MD vaccinations are shown by red brackets with stars.

To evaluate the immune protection efficacy of currently available classical MD vaccines, the second batch of animal experiments was performed using 1-day-old SPF chickens, which were first vaccinated with one of the four distinct commercial MD vaccines (CVI988, HVT, CVI988+HVT, or 814) and then challenged by SDCW01, one of the most virulent MDV isolates, as described above and listed in Table 2. Immune organs from each group were sampled and weighed for calculating the immune organ indices at 14 and 21 dpc, and the body mass of surviving birds from each group was recorded at one-week intervals from 14 to 49 dpc and at 67 dpc. Compared to the CEF mock controls, as shown in Figure 3a, the body masses of SDCW01-challenged birds were lower at all time points post-challenge, with particularly significant differences at 35, 42, 49, and 67 dpc (*p* < 0.01). However, for the MD-vaccinated/SDCW01-challenged groups, no significant differences in body masses were observed compared to the CEF mock group, except for the CVI988-vaccinated birds showing significantly lower body masses at 42 dpc, which was also significantly different from the CVI988+HVT or 814 vaccinated birds.

For the thymus indices, the MD-unvaccinated/SDCW01-challenged group was significantly lower than the CEF mock group at 14 and 21 dpc (*p* < 0.01). However, for the MD-vaccinated/SDCW01-challenged groups, except for the 814-vaccinated group with a significant difference at 21 dpc (*p* < 0.05), the thymus indices of the surviving birds in the other groups measured at both time points had no significant differences compared to the CEF mock group (Figure 3b). No significant difference was observed among the four MD vaccination groups, either. For the bursa indices, the MD-unvaccinated/SDCW01-challenged group was significantly lower than those of the CEF mock group at 14 and 21 dpc (*p* < 0.01), but, for the four distinct MD-vaccinated/SDCW01-challenged groups, no significant difference was observed in the birds that survived compared to the CEF mock controls or to themselves (Figure 3c). Furthermore, the spleen indices of MD-unvaccinated/SDCW01-challenged birds were found to be significantly higher than those of the CEF mock group at 14 and 21 dpc (*p* < 0.05). However, for all the MD-vaccinated/SDCW01-challenged birds, the spleen indices were higher than those of the CEF mock group, especially for those of the CVI988+HVT- and 814-vaccinated groups, with significant differences at 21 dpc (*p* < 0.01) (Figure 3d). Among the four MD vaccines, a significant difference between CVI988 and CVI988+HVT or 814 was observed at 21 dpc (*p* < 0.01), and a similar significant difference was also observed for HVT.

### 3.4. Comparison of the Immune Protection Efficacy of Distinct MD Vaccines against the SDCW01 Strain

After the MD-vaccination and virus challenge, the immune protection efficacy of four different commercial MD vaccines was evaluated. The number of non-specific deaths (*n* = 4, 0, 0, 0, 2, 1 for six groups), which may have been caused by adverse effects of virus inoculation or other factors in the first week, and those euthanized at 14 and 21 dpc for immune organ sampling were excluded from the statistical analysis. All of the other dead and surviving birds were used for calculating the rates of MD morbidity, mortality, tumor incidence, and PIs. As shown in Table 3, no birds in the CEF mock group became sick or died during the whole period of experiment, and both the morbidity and mortality were 0%. However, the morbidity and mortality rates of the CEF-mock-vaccinated/SDCW01-challenged group were 100% and 88.5%, respectively, which were completely consistent with the results of the first batch of animal experiments for the pathogenicity analysis (Table 2). As for the birds separately vaccinated with CVI988, HVT, CVI988+HVT, or 814 vaccines and subsequently challenged by the SDCW01 strain, the morbidities were 53.9%, 61.5%, 50%, and 72%, the mortalities were 46.2%, 11.5%, 41.7%, and 48%, and the corresponding PIs were 46.2%, 38.5%, 50%, and 28%, respectively. Different from the mortality, no significant differences were observed in the morbidities and PIs between different MD vaccination groups when compared to each other (Table 3).

Furthermore, the tumor incidences in dead and surviving birds in each group were statistically analyzed. As shown in Table 4, no deaths or tumors were observed in the CEF mock group during the whole experimental period. During the 67-day challenge test, the tumor incidence of dead birds in the CEF-mock-vaccinated/SDCW01-challenged group was 47.8%, whereas the tumor incidence of dead birds was 0% for the birds in all of the four distinct MD-vaccinated/SDCW01-challenged groups. At 67 dpc, the autopsy observation of all surviving birds showed that the tumor incidence in the CEF-mock-vaccinated/SDCW01-challenged birds was 100%. The tumor incidences in the CVI988 or HVT-vaccinated birds were 14.3% or 13%, respectively, whereas the tumor incidence in both of the CVI988+HVT and 814-vaccinated birds was 0%. Taken together, the cumulative tumor incidence of the CEF-mock-vaccinated/SDCW01-challenged birds was 53.9%, similar to the data obtained from animal experiments for the pathogenicity analysis (Table 2). For MD-vaccinated/SDCW01-challenged birds, the cumulative tumor incidences that occurred in the CVI988, HVT, CVI988+HVT, or 814 vaccination groups were 7.7%, 11.5%, 0%, and 0%, respectively (Table 4).

### 3.5. Survival Curves of MD-Vaccinated Birds Challenged by the SDCW01 Strain

**Figure 4:**
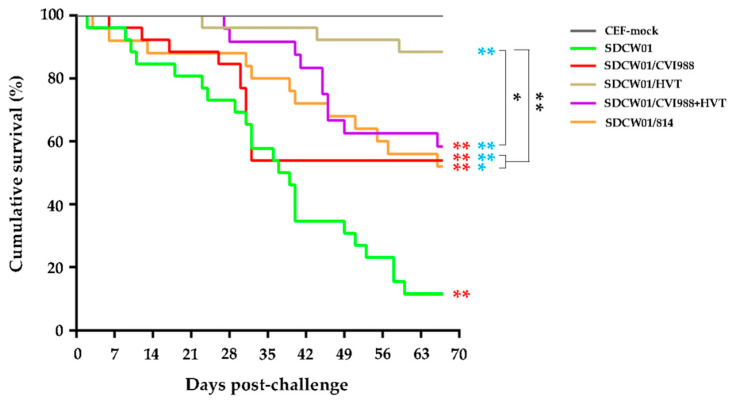
Survival curves of the MD-vaccinated chickens challenged by SDCW01 over the 67-day experimental time period. For each of the experimental groups, 1-day-old birds were separately vaccinated with MD vaccines CVI988, HVT, CVI988+HVT, or 814, containing 2000 PFUs of viruses by subcutaneous injection, whereas two groups of birds were injected with equal volumes of mock CEFs for the mock control and the positive control. At 7 days of age, each of the MD-vaccinated and positive control groups was separately challenged by the SDCW01 virus (1000 PFUs per bird) via intra-abdominal inoculation. For the unchallenged negative controls, equal volumes of mock CEFs were injected via the same route. Early deaths due to adverse effects of intra-abdominal infection and birds sacrificed for body weighting and immune organ collections were excluded from the data analysis. The differences in survival patterns between each virus-challenged group were statistically analyzed with a Log-rank (Mantel–Cox) test (GraphPad Prism Version 6.0). Significant differences compared to CEF mock infection controls are shown by red stars (** *p* < 0.01). The significance of MD-vaccination groups compared to SDCW01-challenge controls are shown by blue stars (* *p* < 0.05 or ** *p* < 0.01), whereas those among distinct MD vaccinations are shown by black brackets and stars.

After the MD-vaccination and virus challenge, the survival curves of birds in each group were drawn according to the daily recorded deaths, except for those of early deaths that occurred in the first week, possibly due to the adverse effects of intra-abdominal infections or other situations. As demonstrated in Figure 4, no deaths were observed in the CEF mock group during the whole experimental time period. In the CEF-mock-vaccinated/SDCW01-challenged group, the birds continuously developed the disease throughout the course of the experiments. However, in the MD-vaccinated/SDCW01-challenged groups, the survival rates of all experimental birds were higher than those of the CEF-mock-vaccinated/SDCW01-challenged group. Disease in 814 vaccinated birds occurred throughout the experiment, and the trend in MD-associated mortality was similar but more moderate than that in the CEF-mock-vaccinated/SDCW01-challenged group. Mortality in CVI988-vaccinated birds mainly occurred at the early stage of the disease (before 35 dpc), whereas it occurred mainly at the middle and late stages of the experiments, during the 3rd to 10th weeks post-challenge, in the HVT- or CVI988+HVT-vaccinated birds. Accumulatively, the survival rates in CEF-mock and SDCW01-challenged controls were 100% and 11.5%, respectively. In MD-vaccination/challenge groups, the survival rates of SDCW01/CVI988, SDCW01/HVT, SDCW01/CVI988+HVT, and SDCW01/814 were 53.8%, 88.5%, 58.3%, and 52%, respectively, over the 73-day experimental time period. The median survival time in the CEF-mock-vaccinated/SDCW01-challenged group was 38 day, while the median survival times in the MD-vaccinated/SDCW01-challenged groups were uncalculated due to the less than half of cumulative deaths during the whole experimental time period. Statistically, as demonstrated in Figure 4, a significant difference was observed between the survival patterns of CEF-mock-vaccinated/SDCW01-challenged birds and the four MD-vaccinated/SDCW01-challenged bird groups (*p* < 0.05 or *p* < 0.01). For the MD-vaccinated groups, the survival pattern of the HVT-vaccinated/challenged group was significantly different than those of the other three MD-vaccinated/challenged groups (*p* < 0.05 or *p* < 0.01), but no difference was observed among the three MD vaccines, including CVI988, CVI988+HVT, and 814.

## 4. Discussion

Frequent outbreaks of MD in vaccinated chicken flocks have recently been reported in many countries, especially in Asia [24]. Although a number of epidemic MDV strains have been isolated from such outbreaks, most of them still lack rigorous experimental validation, and the virulence rates of these isolates still remains unclear. During the period of 2011–2021, at least 191 chicken farms distributed in 17 Chinese provinces experienced MD outbreaks [24], of which MD clinical cases were reported in the provinces of Henan and Shandong (2011–2012), Liaoning, Jilin, and Anhui (2011–2015), and Guangxi, Guangdong, and Yunnan (2017–2020). In recent years, especially since 2019, outbreaks of MD in large areas of China have become more frequent and serious, but the main causes and effective countermeasures remain to be established. In a recent study [28], we performed a systematic epidemiological investigation into avian immunosuppressive and neoplastic diseases covering four provinces in central China during the period of 2020–2022. This study revealed that the current prevalence of virulent MDV in chicken flocks was the main reason for the recent large number of outbreaks of poultry tumor-bearing diseases. Thus, to further reveal the underlying causes of the current MD outbreak and explore effective strategies for the future prevention and control of the disease, we performed the first systematic comparative pathogenicity analysis of the newly isolated MDV strains and further evaluated the immune protection efficacy of current commonly used MD vaccines.

Using 1-day-old white leghorn SPF chickens, the pathogenicity and oncogenicity rates of seven MDV isolates were first determined. The results showed that these isolates, including SDCW01, HNXZ05, HNSQ05, HNSQ01, HNKF01, HNXZ01, and HNFQ03, were all pathogenic to chicken hosts. Sequentially, two strains, SDCW01 and HNSQ01, were displayed as the most virulent, followed by the other two highly virulent strains of HNXZ05 and HNSQ05. However, the remaining three strains of HNKF01, HNXZ01, and HNFQ03 were relatively weak in virulence, and birds infected with HNFQ03 and HNXZ01, which showed death but no gross tumors, were observed during the whole experimental period. Compared to HNKF01, HNFQ03, and HNXZ01, the strains of SDCW01, HNXZ05, HNSQ05, and HNSQ01 with high virulence rates also caused severe atrophy of the thymus and bursa in virus-infected chickens. This indicated that these MDV strains also exerted significant immunosuppressive effects on chicken hosts, which was consistent with the early death of virus-infected birds reflected by the survival curves. These data also suggest co-epidemics of MDV strains with variable virulence rates in Chinese chicken flocks. More importantly, compared to those MDV strains isolated ten years ago [10], HV-MDV strains such as SDCW01 and HNSQ01 that can cause 100% MD incidences and more than 50% tumor occurrences have become more prevalent in chicken flocks.

In the subsequent animal experiment, SDCW01, one of the most virulent MDV strains presently identifiable by pathogenicity analysis, was chosen to challenge the MD-vaccinated birds for evaluating the immune protection efficacy of different MD vaccines, CVI988, HVT, CVI988+HVT, and 814. The results of this study showed that none of the four classical MD vaccines were able to provide an ideal immune protection against the challenge of SDCW01. The PI of the four MD vaccines was no more than 50%, and the lowest one had only 28%, but no significant differences were observed among the different MD vaccines. It seems that the cumulative mortality and survival curves of the HVT-only vaccination were superior to both CVI988 and the bivalent vaccine. It is well known that the HVT virus replicates faster than CVI988 and may cause better innate immune responses, and it had the best survival curve during the 63-day experiment time period. However, we also observed that the shedding of chicken tail feathers caused by virus release was more severe in HVT-vaccinated birds at about 30 days post-SDCW01-challenge. Furthermore, the birds vaccinated with monovalent CVI988 or HVT vaccines still developed tumors, with cumulative tumor incidences of 7.7% and 11.5%, respectively. Taken together, it seems that CVI988+HVT, rather than HVT, provided a better immune efficacy against SDCW01. Unfortunately, due to COVID-19 in 2022, we were quarantined in our communities and had to terminate the virus-challenge experiments ahead of the planned 90-day experimental time period. If the experiments could have proceeded as expected, we speculate that the occurrences of tumors may have been increased and have finally led to an even lower PI value in the HVT-vaccinated birds. Our data indicated conclusively that the currently circulating HV-MDV strains have significantly broken through the immune protection provided by classical MD vaccine strains authorized and most widely used in China, including CVI988, HVT, and 814.

In recent years, the increased virulence of epidemic MDV strains and the decreased efficacy of MD vaccines may have been the most important factors responsible for the frequent outbreaks of MD. In fact, such a phenomenon may not be a surprise. As early as ten years ago [12], we isolated the HV-MDV variant HN302 (originally named HNLH302) from MD-vaccinated chickens in central China. In the follow-up study [10], we revealed that neither CVI988 nor HVT could provide satisfactory immune protection against HN302, and the PIs were only 27% and 33.3%, respectively. These data suggested that the HV-MDV variants had been prevalent for a long time in China. In the present work, for the choice of suitable MDV isolates for animal experiments, we first performed sequencing and a phylogenetic analysis of the *meq* genes of 15 new MDV isolates derived from the MD-vaccinated chicken flocks suffering with clinical MD cases with tumors previously reported [28]. To further reveal the major determinants responsible for the increased virulence of HV-MDV Chinese variants, *meq* genes cloned from hundreds of clinical samples and all the other MDV isolates were sequenced, and all the MDV isolates used in this study were also sent out for whole genome sequencing. Once this had been finished, one of the series of studies we have planned, an overall phylogenetic analysis of these viruses prevalent in China in most recent years, will be performed. This will possibly provide more important clues for revealing the underlying determinant responsible for MD immune failure.

In the past decade, the virulence of MDV epidemic strains in China has continuously evolved, and the immune efficacy of MD vaccines has gradually decreased. In 2012, a virulent MDV strain, SD2012-1, isolated in the Shandong province of China, was able to break through the immune protections of HVT and HVT+SB-1 vaccines, for which the PIs were only 5.1% and 14.7%, respectively [32]. In 2011, the PIs provided by CVI988 against the epidemic MDV strains isolated from Liaoning and Jilin provinces of China, such as LCC, LLY, and LTS, could still reach 85.7%, 92.3%, and 66.7%, respectively [9]. The MDV strains ZY/1203 and WC/1110 were isolated almost at the same time, from Guangdong and Heilongjiang, and the PIs provided by the CVI988 vaccine also reached 82.4% and 83.3%, respectively [13]. Subsequently, in 2013, a strain of MDV, SX1301, was isolated in Shanxi province from a clinically diseased chicken, and the protection rate of the CVI988 vaccine was still able to reach 83% [33]. In general, the CVI988 vaccine could provide a good immune protection for most MDV epidemic strains in China before 2015. However, since then, the PIs provided by CVI988 and 814 against the MDV isolate, such as BS/15 isolated from Jilin province in 2015, were only 33.3% and 66.7%, respectively [34]. Similarly, the PIs provided by the CVI988 and 814 vaccines against another MDV strain, GX18NNM4, isolated in 2018 from Guangxi province, were only 39.9% and 61.1% [35]. Up until this study, all the data suggested that classical MD vaccine strains such as HVT, CVI988, SB-1, and 814 were not able to provide ideal immune protection for most of the new MDV isolates in China. Undoubtedly, some of the HV-MDV variants, such as HN302 and SDCW01, significantly broke through the immune protection of classic MD vaccines. For future work, the development of novel efficient MD vaccines for the challenge of dealing with new emerging HV-MDV strains and variants is urgent.

It is well known that *meq* is a major oncogene responsible for the development of MD lymphomas, and the knock-out of the *meq* gene from the viral genome can lose the oncogenicity of MDV [4]. As the most important pathogenic factor, point mutations have frequently occurred in the *meq* gene, accompanying persistent virus evolution [24,36]. Although it is currently unclear which mutations, in which specific loci are directly related to the increased virulence of the virus, replacing the *meq* genes between different pathotypes of MDV strains, such as CVI988 (mMDV), JM\120W (vMDV), RB-1B (vvMDV), and N (vv+MDV), can significantly alter the virulence of recombinant MDV strains [37]. Previously, many scholars have tried to use the virulent MDV-1 strain as a parental virus to develop novel MD vaccines, e.g., using the vvMDV strain Md5 to develop a *meq*-deleted MD vaccine using a bacterial artificial chromosome (BAC) and homologous recombination technology [38]. In China, two MD vaccine strains with *meq*-deletion have been successfully developed. One is SC9-1, generated by knocking out the *meq* gene from the viral genome of GX0101 (a vvMDV strain isolated from Guangxi province in 2001) and provides a better immune protection effect against Md5 than CVI988 [39,40]. The other one is rMSΔmeq, which was developed using another Chinese MDV strain, LMS (isolated from south-west China in 2007), as its parental virus; this could effectively protect against the disease induced by Md5 and some of the virulent MDV variants, such as LCC and LTS [41]. Previous studies have shown that both GX0101 and LMS were clustered in the same genetic evolution branch as the vast majority of MDV strains isolated in China in recent years [36]. From the perspective of MDV antigenicity, MD vaccines generated from these parental strains may provide better immune protection for the HV-MDV variants (e.g., SDCW01 and HN302) currently prevalent in China, which is worthy of further exploration in future studies. Furthermore, only one of the HV-MDV strains has been chosen for evaluating the immune protection of classical MD vaccines at present. In future studies, it will be worth testing more vaccine products, especially the newly developed MD vaccines such as the *meq* gene-deleted vaccine, and using more HV-MDV strains such as HNXZ05, HNSQ05, and HNSQ01 for the evaluation of the immune protection efficacy of MD vaccines, in order to provide more important references for the efficient control of the disease.

## Figures and Tables

**Table 1:** Background of seven MDV isolates used in this study.

No.	Strain Abbreviation	Original Signature Used for MDV Isolates ^a^	Year and Month	Source ^b^	Host	Ages for Virus Isolation (Days)	Passages on CEF
1	SDCW01	SD-CW-01-C3	2021, May	SDCW	Hy-Line Brown	70	6
2	HNXZ05	HN-XZ-XZZ-05-C1	2021, May	HNXZ	Partridge chicken	80	5
3	HNSQ05	HN-SQ-YC-ZL-05-C2	2021, May	HNYC	Jinghong	65	6
4	HNSQ01	HN-SQ-ZC-DW-01	2021, May	HNZC	Jinghong	70	6
5	HNKF01	HN-KF-WS-01-C2	2021, September	HNWS	Muyuan Red	70	5
6	HNXZ01	HN-XZ-XZZ-01-C4	2021, May	HNXZ	Partridge chicken	80	5
7	HNFQ03	HN-FQ-YJ-03-C1	2021, September	HNFQ	Hy-Line Brown	70	5

^a^ Original signature and method used for the designation of new MDV isolates during the processes of virus isolation, e.g., SD-CW-01-C3, SD = Shandong Province, CW = Chengwu County, 01 = No. 1 of diseased birds, C3 = the third clone of purified MDV plaques on CEF monolayers in 6-well plates for virus isolation. ^b^ Geographical location is displayed as abbreviated province plus city/county. SD, Shandong province; HN, Henan province; CW, Chengwu county; XZ, Xinzheng county; YC, Yucheng county; ZC, Zhecheng county; WS, Weishi county; FQ, Fengqiu county.

**Table 2:** Cumulative morbidity, mortality and gross tumor incidence in birds infected with different MDV strains calculated at 73 days post-infection (dpi).

No.	Strains	Total Numbers ^A^	Diseased Birds	Morbidity (%) ^B^	Deaths	Mortality (%) ^C^	Gross Tumors	Tumor Incidence (%) ^D^
1	SDCW01	30	30	100.0 ^a b c d^	25	83.3 ^a b c d^	15	50.0 ^a b c^
2	HNSQ01	30	30	100.0 ^a b c d^	26	86.7 ^a b c d e^	19	63.3 ^a b c d e f^
3	HNXZ05	30	28	93.3 ^a b c d^	22	73.3 ^a b c d^	10	33.3 ^a b c^
4	HNSQ05	30	27	90.0 ^a b c d^	18	60.0 ^a b d^	9	30.0 ^a b c^
5	HNKF01	33	15	45.5 ^a b^	7	21.2 ^a^	8	24.2 ^a b c^
6	HNFQ03	29	10	34.5 ^a^	10	34.5 ^a^	0	0
7	HNXZ01	30	3	10.0	3	10.0	0	0
8	CEF mock	26	0	0	0	0	0	0

^A^ Total number = number of virus-infected birds in each group − total number of birds necropsied for weighting − number of non-pathological death. ^B^ All birds showing clinical MD symptoms, dead and surviving ones with gross tumors were classified as MD cases. Morbidity (%) = number of MD cases/total number × 100. ^C^ Mortality (%) = number of deaths/total number × 100. ^D^ Tumor incidence (%) = number of deaths or survivals with gross tumors/total number × 100. The statistical significances of morbidity, mortality, and tumor incidence between different groups were compared using Z test. ^a b c d e f^ Indicate the significant differences compared to CEF mock, HNXZ01, HNFQ03, HNKF01, HNSQ05, and HNXZ05, respectively.

**Table 3:** Cumulative morbidity, mortality and PIs of distinct MD-vaccinated chickens challenged by the SDCW01 strain calculated at 67 days post-challenge (dpc).

Vaccines *	Strains	Total Numbers	Deaths	Mortality (%)	Diseased Birds ^#^	Morbidity (%)	PI (%)
CEF-mock	CEF-mock	22	0	0	0	0	/
CEF-mock	SDCW01	26	23	88.5 ^a^	26	100.0 ^a^	/
CVI988	SDCW01	26	12	46.2 ^a b^	14	53.9 ^a b^	46.2
HVT	SDCW01	26	3	11.5 ^b c^	16	61.5 ^a b^	38.5
CVI988+HVT	SDCW01	24	10	41.7 ^a b d^	12	50.0 ^a b^	50.0
814	SDCW01	25	12	48.0 ^a b d^	18	72.0 ^a b^	28.0

* Equal volume of mock CEF suspension serves as MD vaccine negative controls. ^#^ Surviving birds showing gross tumors inspected at 67 dpc were also counted as MD cases. “PI” means the protection index. “/” means not applicable. The statistical significance of morbidity, mortality, and PIs between different groups was compared using Z test. ^a b c d^ Indicate the significant differences compared to CEF mock, SDCW01, CVI988, and HVT, respectively.

**Table 4:** Cumulative gross tumor incidences in distinct MD-vaccinated chickens challenged by the SDCW01 strain calculated at 67 days post-challenge (dpc).

Vaccines *	MDV Strains *	Total Birds	Deaths		Survivals		Total	
			Tumors /Deaths	Tumor Incidence (%)	Tumors /Survivals	Tumor Incidence (%)	Tumors	Tumor Incidence (%)
CEF-mock	CEF-mock	22	0/0	0	0/22	0	0/22	0
CEF-mock	SDCW01	26	11/23	47.8	3/3	100.0 ^a^	14/26	53.9 ^a^
CVI988	SDCW01	26	0/12	0 ^b^	2/14	14.3 ^b^	2/26	7.7 ^b^
HVT	SDCW01	26	0/3	0	3/23	13.0 ^b^	3/26	11.5 ^b^
CVI988+HVT	SDCW01	24	0/10	0 ^b^	0/14	0 ^b^	0/24	0 ^b^
814	SDCW01	25	0/12	0 ^b^	0/13	0 ^b^	0/25	0 ^b^

* Equal volume of mock CEF suspension served as MD vaccine negative control. The statistical significance of tumor incidence between different groups was compared using Z test. ^a b^ Indicate the significant differences compared to CEF mock and SDCW01, respectively.

## Data Availability

Not applicable.
